# Geometric evaluation of a deep learning method for segmentation of urinary OARs on magnetic resonance imaging for prostate cancer radiotherapy

**DOI:** 10.1016/j.ctro.2025.101091

**Published:** 2025-12-05

**Authors:** Jennifer Le Guévelou, Miguel Castro, Blanche Texier, Anaïs Barateau, Romane-Alizé Martin, Caroline Lafond, Igor Bessières, Jean-Claude Nunes, Renaud De Crevoisier, Oscar Acosta

**Affiliations:** a Department of Radiation Therapy, Centre Eugène Marquis, Rennes, France; b Laboratoire du Traitement de l’image (LTSI-UMR 1099), Université de Rennes, France; c Department of Physics, Centre Eugène Marquis, Rennes, France; d Department of Physics, Centre Georges François Leclerc, Dijon, France

**Keywords:** Automatic segmentation, Deep learning, Intraprostatic urethra, Urinary organs at risk, Magnetic resonance imaging

## Abstract

**Introduction:**

While urinary organs at risk (OARs) such as the intraprostatic urethra and the bladder trigone are increasingly recognized as associated with severe genitourinary toxicity, their delineation in clinical practice is time consuming and probably associated with a large interobserver variability. The aim of this study was to propose a magnetic resonance (MR) deep learning segmentation of urinary OARs for prostate cancer (PCa) radiotherapy (RT), based on a validated atlas.

**Material and methods:**

In this multicentric study, a convolutional neural network (CNN) for image segmentation (nnU-Net) was trained and validated on three image datasets. Two datasets came from MR-linac devices (Unity®, Elekta and MRIdian®, Viewray), and one dataset came from the PROSTATEx database (MAGNETOM® Trio and Skyra, Siemens). Evaluation of the deep learning segmentation was performed using dice score coefficients (DSC), surface distance (SD) and Hausdorff distance.

**Results:**

A total of 265 MRI were analyzed. The mean DSC for all urinary structures was 0.88. The automatic segmentation model proved to be effective in the segmentation of the target volume and large OARs such as the bladder (mean DSC ranging of 0.95). Regarding urinary OARs, the mean DSC ranged between 0.50 and 0.68. The Hausdorff distance ranged between 4.0 mm to 10.3 mm for urinary OARs, highlighting local mismatches caused by large anatomical variations between patients. However, the SD ranged between 1.0 mm and 1.3 mm for urinary OARs, highlighting an overall good surface correlation for all organs.

**Conclusion:**

This multicentric study is the first to propose a nnU-Net deep learning model for the delineation of urinary OARs, that can be applied to various image dataset. Further work is needed to assess the dosimetric impact of such variations, in various clinical scenarios.

## Highlights

• This multicentric study is the first to provide a deep learning method for segmentation of urinary OARs.
• The mean DSC for urinary OARs ranged between 0.50 and 0.68.
• The mean Hausdorff distance for urinary OARs ranged between 4.0 mm to 10.3 mm.
• The mean surface distance for urinary OARs ranged between 1.0 and 1.3 mm.

## 1 Introduction

External beam radiotherapy (EBRT) represents one of the standard of care approach for the management of localized prostate cancer (PCa) [1]. Technological improvements of the past decade with the implementation of both intensity-modulated radiotherapy (IMRT) and image-guided radiotherapy (IGRT) enabled to reduce both acute and late severe genitourinary (GU) toxicity [2]. The development of new irradiation strategies for the management of PCa such as “augmented RT” with either increase in dose delivered to the prostate gland or increase in target volume have demonstrated its ability to improve oncological outcomes, however can come at the cost of increased GU toxicity [3]. Also, prostate reirradiation is increasingly proposed within prospective clinical trials for radiorecurrent PCa, but showed a high-risk of late and severe GU toxicity [4,5].

Various dose-constraints on the bladder and bladder wall are implemented in case of PCa EBRT planning [6]. However, concerns have been raised on the role of additional urinary structures in the development of late GU toxicity, namely: the ureters [7], the bladder trigone [[8], [9], [10]], the transurethral resection of prostate (TURP) cavity, and the intraprostatic urethra [[11], [12], [13]]. To date, comparisons between trials are hampered by the variability of definitions of these structures between studies. Recently, the Francophone Group of Urological Radiation Therapy (GFRU) provided a magnetic resonance imaging (MRI) expert consensus on the delineation modalities for urinary organs-at-risk (OARs) [14]. However, the delineation of these structures is time-consuming and probably subjected to interobserver variability. Only few studies were led to assess interobserver variability regarding the delineation of urinary OARs. In the study led by Ong et al., the mean dice similarity coefficient (DSC) for the intraprostatic urethra was 0.62 [15]. Automatic segmentation represents a promising way to reduce interobserver variability for the delineation of urinary OARs.

Several approaches have been developed in order to perform automatic segmentation. Atlas-based methods represent the historical approach, however are limited when complex and small organs are considered [16]. Machine learning-based methods represent a category of algorithm that enables automatic learning without prior programming, and are a promising approach for automatic segmentation. Among the machine learning methods, deep learning approaches using convolutional neuronal networks (CNN) represent a subtype of machine learning that does not need human intelligence to highlight the relevant features, but instead learns from data placed within a “training set”. Specifically, nnU-Net is a deep learning method that allows for optimization of all the different steps (preprocessing, network architecture, data augmentation, training and post-processing), for any given task. The nn-Unet CNN shows a high accuracy for segmentation tasks, and enables a tridimensional analysis of the structures [17].

This work aimed to evaluate deep learning segmentation using an nn-Unet CNN of urinary OARs for PCa EBRT.

## 2 Material and methods

### 2.1 Databases

MRI were obtained from two different MR-linac devices, enabling magnetic resonance guided radiotherapy (MRgRT). The first dataset was obtained from the MRIdian (ViewRay System, Denver, USA) radiotherapy device, combining a 0.35 Tesla MRI and a 6 MV FFF linear accelerator enabling step-and-shoot intensity modulated radiotherapy (IMRT). The planning images were true fast imaging with steady state free precession (TRUFI) sequences, with a T2/T1 weighted image contrast. The second dataset was obtained from the Unity (Elekta®), combining a 1.5 Tesla MRI and a 7 MV FFF linear accelerator enabling step-and-shoot IMRT. The planning images were T2-weighted spin echo images.

A total of 200 MRI from the PROSTATEx challenge were also added to the database [18,19], in order to both obtain a segmentation model that is efficient across a wide range of MRIs, as well as increasing the number of MRI within our database. MRI were obtained from diagnostic 3 T Siemens devices (MAGNETOM Trio and Skyra). The planning images were T2-weighted spin echo images.

Exclusion criteria for this study were: patients that have received a radical prostatectomy, patients with a previous history of transurethral resection of the prostate (TURP), patients with completely empty bladder.

### 2.2 Delineation of urinary OARs

The following structures were delineated: prostate, ureters, bladder neck, bladder trigone, bladder, intraprostatic urethra, striated sphincter, bulbous urethra, membranous urethra, based on the consensus published by the GFRU [14]. MRI from the Unity and MRIdian database were delineated by the same experienced radiation oncologist (JLG), that wrote the consensus. MRI coming from the PROSTATEx challenge were implemented within the database, with structures that were previously validated by experts in radiology and urology, i.e the prostate and intraprostatic urethra.

### 2.3 Preprocessing

Preprocessing was performed in order to homogenize MRI, that were obtained from several devices, and consisted in several consecutive steps. MRI non-uniformity was corrected through the use of both a N4 bias field correction [20], an histogram matching and a filtering by gradient anisotropic diffusion [21]. MRI were cropped at a maximum 12 cm above and under prostate isocenter, in order to have a common field of view (FOV). This methodology has already been described by Texier *et al* [22].

### 2.4 Training

In order to train the segmentation network, we employed a 5-fold cross-validation strategy, where in each fold 80 % of the data were used for training and 20 % for testing. Then, the segmentation network was trained to delineate urinary OARs, using nnU-Net CNN (open-source solution). The nn-Unet was used generating a 3D configuration, with full image resolution. As input, we used the MRI and the structures delineated by the radiation oncologist as well as structures provided within the public repository PROSTATEx.

The model was trained using the DC_and_CE loss function from the nnU-Net framework, which combines Dice loss (DC) and cross-entropy (CE) loss with respective weights of 0.7 and 0.3. This combined loss is used as the default because it balances robustness in learning small or complex structures (Dice) with stable gradient flow and classification accuracy (CE), providing improved segmentation accuracy and robustness in medical imaging [23]. Specific modifications were made to specific parameters to the nnUnet CNN. Class-specific weighting was applied within the loss function, assigning higher weights to small structures to further enhance their segmentation performance. An aggressive oversampling strategy and patch center jittering were implemented to increase the representation of these classes during training. Optimization was performed using the Adam optimizer with an initial learning rate of 10^-4^. To improve generalizability and robustness of the model, a 5-fold cross-validation framework was employed, with each fold trained for up to 1,000 epochs.

Following the 5-fold cross-validation, the final model was further evaluated on an independent validation set of 10 cases to ensure robustness and applicability across a broad spectrum of MR devices. After analyzing the initial results of automatic segmentation and observing fragmented delineation of the intraprostatic urethra (attributable to its small diameter), a post-processing step was applied to enforce continuity of urethral segments along the centerline [24].

Trained model and scripts have been made available for use in Github (https://github.com/LTSI-U1099/urinary-OARs-segmentation-nnUNet).

### 2.5 Evaluation

**Fig. 1:**
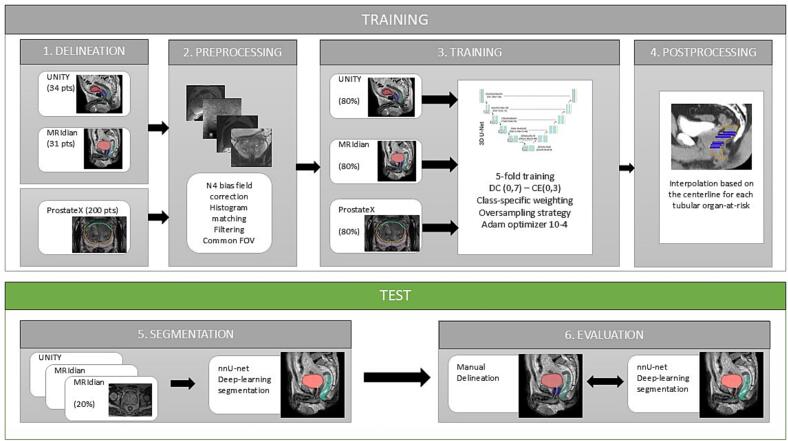
Workflow of the study.

The segmentation network was evaluated using several metrics (Fig. 1). Volumetric Dice Score Coefficient (DSC) is a similarity metric that represents the overlap between predicted and “ground truth” segmentation, and ranges from 0 to 1, with a higher score being associated with a greater overlap. Based on previous studies [25,26] and considering the exceedingly small size of urinary OARs, a volumetric DSC of 0.5 was deemed as significant for urinary OARs segmentation. We also assessed the Hausdorff distance (HD), which is a difference metric enabling to assess the locations in the segmented structures which matches the “ground truth”. The HD is defined as the distance from a point in the first set to a nearest point in the other one. Surface distances (SD) have also been evaluated, and represent the average of all the distances from points on the boundary of machine segmented region to the boundary of the ground truth. SD is expressed in mm. The Jaccard index was not assessed for the segmentation of urinary OARs, due to its high sensitivity to small errors, resulting in disproportionately harsh penalties for small segmentation deviations [27]. Also, because the Jaccard index is a strict overlap metric, it might reflect poorly clinical relevance of segmentation errors in small anatomical structures (where small spatial deviations may be tolerable).

## 3 Results

A total of 77 patients from the Unity and MRIdian databases were assessed for this study. A total 12 patients received a TURP for prostate benign hyperplasia, and were further excluded from the study, leaving 65 patients eligible for the study. Additionally, 200 patients coming from the PROSTATEx database were added. Specifications regarding the database is provided in Table 1.

**Table 1:** Specification of the databases for organs-at-risk segmentation.

Center	Number of patients analyzed	MR sequence	Resolution (mm)	TR/TE (ms)	Field-of-view (mm)	Patients with TURP cavity	Number of patients included in the study
MRIdian database (Viewray)	39	TRUFI sequences (0.35 T)	In plane: 1.5 mm × 1.5 mm Slice thickness: 1.5 mm	3.4/1.4	448 × 300	8	31
Unity database (Elekta)	38	T2 weighted spin echo sequences (1.5 T)	In plane: 1 mm × 1 mm Slice thickness: 1 mm	1400/182	350 × 350	4	34
PROSTATEx challenge database (Siemens)	200	T2 weighted sequences (3 T)	In plane: 0.5 × 0.5 mm Slice thickness: 3.6 mm	2200/120	280 × 280	0	200

Abbreviations: MRI = magnetic resonance imaging, TURP = transurethral resection of the prostate, TRUFI = true fast imaging with steady state free precession, T = Tesla.

### 3.1 Volume of the structures

Table 2 shows volumetric data on the structures of interest. The largest OAR was the bladder, with a mean volume reaching 192 cc, followed by the prostate gland with a mean volume of 35 cc, respectively. Other urinary OARs were much smaller, with mean volumes ranging between 3 cc (bladder neck) and 0.3 cc (membranous urethra).

**Table 2:** Mean values and standard deviations for similarity metrics for target volume (prostate) and for each organ at risk.

	Ground truth: Mean volume in cm^3^ *(SD)*	Automatic segmentation: Mean volume in cm^3^ *(SD)*	Mean volumetric DSC (SD)	Mean surface Distance in mm (SD)	Mean Hausdorff distance in mm (SD)
Prostate	39.8 (±7.3)	36.2(±9.2)	0.85(±0.07)	1.6(±0.61)	7.4(±2.20)
Bladder	192.8(±103)	188.0(±97.6)	0.95(±0.01)	0.9(±0.2)	7.5(±2.65)
Intraprostatic urethra	1.0(±0.21)	1.0(±0.18)	0.53(±0.1)	1.2(±0.21)	4.7(±1.22)
Ureters	2.5(±0.84)	3.7(±0.98)	0.68(±0.17)	1.3(±1.0)	10.3(±5.68)
Bladder neck	3.7(±1.11)	5.0(±1.34)	0.64(±0.14)	1.3(±0.63)	8.5(±3.57)
Bladder trigone	2.0(±0.87)	1.9(±0.84)	0.50(±0.1)	1.1(±0.4)	7.1(±2.82)
Bulbous urethra	0.4(±0.09)	0.4(±0.15)	0.54(±0.05)	1.1(±0.29)	8.7(±5.48)
Membranous urethra	0.3(±0.08)	0.3(±0.11)	0.57(±0.18)	1.0(±0.43)	4.0(±1.31)

Abbreviations: DSC = dice score coefficient, SD = standard deviation, cm**^3^** = cubic centimeter.

### 3.2 Metrics

**Fig. 2:**
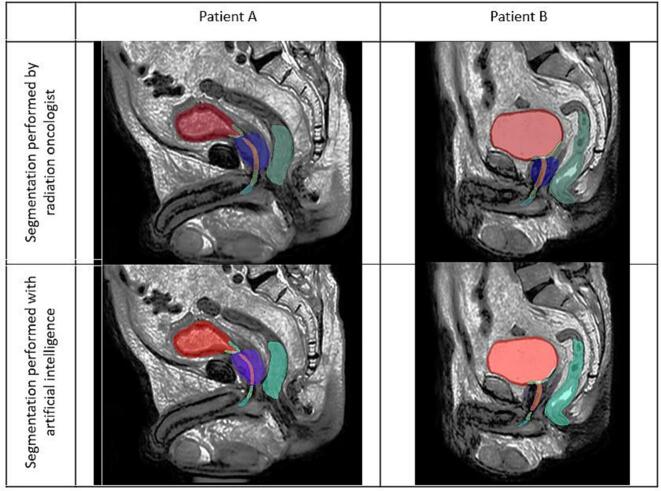
Example of delineation performed by an experienced radiation oncologist and automatic segmentation, in two patients with distinct anatomical patterns. A: patients with a bladder moderately filled (130 cm^3^), and a prostate volume of 23 cm^3^. B: patient with a full bladder (380 cm^3^), and a prostate volume of 37 cm^3^.

An illustration of the automatic segmentation is provided in Fig. 2, displaying two patients with different anatomy (both regarding prostate size, and bladder filling).

**Fig. 3:**
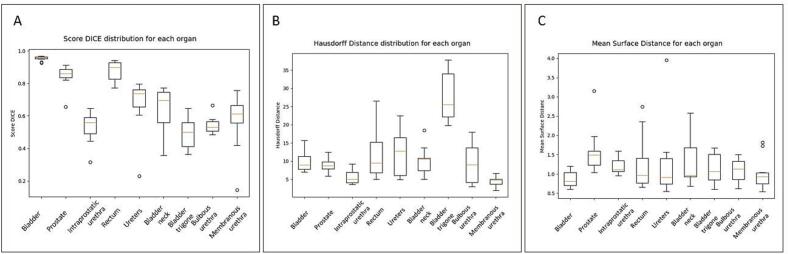
Results of various metrics used for the assessment of the quality of the automatic segmentation model. A: volumetric DICE (%) and interquartile ranges for each organ. B: Hausdorff distance (mm) and interquartile ranges for each organ. C: mean surface distance (mm) and interquartile ranges for each organ.

Regarding the target volume, the DSC for the automatic segmentation of the prostate gland reached up to 0.85 (Table 2) (Fig. 3A). The mean DSC for the bladder reached 0.95. Regarding urinary OARs, all DSC values were superior to 0.5.

The HD exceeded 10 mm for only one organ (the ureters) (Fig. 3B). The lowest HD was demonstrated for the membranous urethra (4 mm), probably due to its fixed position within the genitourinary diaphragm.

When considering the mean SD, variations ranged between 0.9 mm (bladder) to 1.6 mm (prostate) (Fig. 3C), highlighting the fact that even if significant local mismatches exist between the ground truth and automatic segmentation, an overall good surface agreement is demonstrated for a large portion of urinary structures.

## 4 Discussion

This multicentric study is the first to assess a deep learning-based segmentation of urinary OARs on MRI for PCa RT. Also, its originality relies on the fact that it is based on images extracted from two different MR-linac devices as well as one diagnostic device, and therefore the algorithm designed by our team can be applied to both low-field (0.35 T) and high-field (1.5 T or 3 T) MRI. The automatic segmentation was compared with the gold standard (defined by the radiation oncologist) through the use of several metrics, assessing both similarities and differences between the “ground truth” and the automatic segmentation. We obtained overall good surface and DSC for all urinary OARs, highlighting an excellent surface correlation between the ground truth and automatic segmentation. However, the HD was relatively poor for all structures partly due to the large variety of patient’s anatomy in our study.

A limited number of studies implemented urinary OARs in their algorithm for automatic segmentation of prostate MRI, and all algorithm for automatic segmentation were based on a single MR device. Belue et al. obtained a mean DSC reaching 0.61 for the intraprostatic urethra, in dedicated model based on T2-weighted MRI [28]. In another study, Elguindi et al. reported a mean volumetric DSC reaching 0.69 for the automatic segmentation of the urethra [29]. Off note, these results appear to be similar to the interobserver variability reported in the study led by Ong et al [30]., suggesting that automatic segmentation is associated with little benefit regarding In the study led by Xu et al., the volumetric DSC for the urethra reached 0.77 [31]. These results were obtained through the use of a novel post-processing strategy specifically designed for tubular structures (slice-by-slice simple exponential smoothing), allowing to construct a prediction of the current slice from an exponentially weighted average of past observations. The choice of post-processing implemented within our study was motivated following the observation of fragmented urethral segmentation due to its small diameter. We therefore chose to implement a post-processing strategy that was previously validated by our team, allowing for an interpolation of the urethral segments based on its centerline [24]. While these results appear to be superior than ours, it is to be noted that the complexity of our model comes from the segmentation of large number of small-sized urinary OARs. Last but not least, the strength of our study comes from the fact that delineation of urinary OARs has been performed according to validated guidelines [14], which is not the case for other segmentation models.

DSC represents the most frequently used metric for contour agreement. However, there are several limitations that should be kept in mind when it comes to interpretating DSC. DSC values deemed as significant vary in the literature, and are often ranging between 0.5 [25] to 0.7 [26], with the cut-off for significance depending on the clinical situation. For example, when delineating a tumor, a DSC at 0.7 is usually considered insufficient, as it leaves 30 % of the tumor outside the delineated volume. However, for an organ at risk, it can be considered as being satisfactory for RT treatment planning if the structure is usually not spared and/or is very small. In our study, urinary OARs that were delineated are exceedingly small, often with a volume of less than 1 cc. DSC should therefore be accompanied by additional metrics evaluating differences between the delineated and the automatically segmented structures, such as SD and/or Hausdorff distance [32,33]. SD provides additional information such as the surface fraction that needs to be redrawn by the radiation oncologist, and penalizing small structures less than DSC. In our study, SD were excellent, with mean values ranging between 0.9 to 1.6 mm. It must be kept in mind that volumetric DSC and boundary-based metrics often show few to no correlation owing to the fact that they measure different error characteristics. Indeed, DSC represents a global volumetric metric, with small boundary errors being associated with an insignificant impact especially for large structures, while boundary metrics are sensitive to local contour errors. Therefore, a segmentation might match volume well (with a high DSC), but have boundary irregularities resulting in poor HD. Another approach for small tubular structures might also be to determine the centerline distance, which represents the Euclidean distance of ground truth centerline and the centerline of the automatically segmented structure. This approach has already been validated for small tubular structures such as the urethra [34], and enables to analyze the percentage of segmented centerline lying within a certain radius from the “ground truth” centerline. This metric also enables the quantification of the percentage of points lying inside a region around the centerline ground truth, and therefore can provide spatial information on the regions where the centerlines do not coincide, which is of crucial importance for radiotherapy treatment planning.

Although our model demonstrated acceptable SD, large variabilities still exist regarding HD, highlighting the need to refine our model in the future with larger database providing a wider range of anatomical variations. Indeed, even small discrepancies between the structures automatically segmented and the ground truth can result in large variations when considering the dose received by these organs – especially when stereotactic body radiotherapy with sharp dose gradients is performed. Further developments will be led in order to increase the accuracy of our model, including the addition of MRI datasets from new patients from our center. As MRgRT enables to perform repeated MRI before and after each session, the addition of multiple MRI per patients was considered but deemed as non-optimal, as it might have led to an overfitting of the model. Future work will also be performed in order to assess the dosimetric impact of such variations regarding urinary OARs.

Delineation of urinary OARs represents an innovative approach to treatment planning for PCa RT. Several data suggested a dose–response relationship between urinary OARs and especially the intraprostatic urethra and the occurrence of severe GU toxicity [35]. However, no dose constraint has yet been formally validated for PCa RT [36]. Thus, the implementation of this atlas in daily practice and in clinical trials is crucial to make advances in this field. While these structures are small and complex, and most clinicians have little to no experience regarding their delineation, a wide interobserver variability in the delineation of urinary OARs is expected. An evaluation of interobserver variability in urinary OARs delineation is currently ongoing, with the Francophone Group of Urological Radiation Therapy. This study will enable us to obtain a STAPLE (median delineation between expert's) with the aim of presenting concrete examples with different anatomical variations. These efforts will hopefully enable high consistency in the delineation of urinary OARs between experts and centers.

This study is hampered in several ways. First, there might be too few MRI to perform an accurate segmentation of urinary OARs, especially in patients with anatomical variations and/or large prostate gland. While this model will be refined in the near future, it provides the firsts steps towards automatic segmentation of urinary OARs for PCa MRgRT. Also, a validation cohort was not included within this study, which may limit our conclusions regarding the robustness of our model.

## 5 Conclusion

The nnU-Net deep learning model enables a successful segmentation of both target volume and large OARs such as the bladder. An overall good volumetric DSC was obtained for all urinary OARs, and these findings were confirmed by mean SD. Efforts now have to focus on assessing the dosimetric impact of such geometric variations in different clinical scenarios.

## CRediT authorship contribution statement

**Jennifer Le Guévelou:** Conceptualization, Data curation, Formal analysis, Investigation, Methodology, Project administration, Validation, Visualization, Writing – original draft, Writing – review & editing. **Miguel Castro:** Data curation, Formal analysis, Methodology, Writing – review & editing. **Blanche Texier:** Data curation, Formal analysis, Investigation. **Anaïs Barateau:** Writing – review & editing. **Romane-Alizé Martin:** Writing – review & editing. **Caroline Lafond:** Writing – review & editing. **Igor Bessières:** Writing – review & editing. **Jean-Claude Nunes:** Writing – review & editing. **Renaud De Crevoisier:** Writing – review & editing. **Oscar Acosta:** Methodology, Project administration, Writing – review & editing.

## Funding

None.

## Declaration of competing interest

The authors declare that they have no known competing financial interests or personal relationships that could have appeared to influence the work reported in this paper.
